# Behavioral-dependent recursive movements and implications for resource selection

**DOI:** 10.1038/s41598-023-43907-z

**Published:** 2023-10-03

**Authors:** Nicholas W. Bakner, Bret A. Collier, Michael J. Chamberlain

**Affiliations:** 1 Warnell School of Forestry and Natural Resources, University of Georgia, Athens, GA 30602 USA; 2 School of Renewable Natural Resources, Louisiana State University Agricultural Center, Baton Rouge, LA 70803 USA

**Keywords:** Behavioural ecology, Forest ecology

## Abstract

Within home ranges, animals repeatedly visit certain areas. Recursive movement patterns are widespread throughout the animal kingdom, but are rarely considered when developing resource selection models. We examined how behavioral state-dependent recursive movements influenced reource selection of eastern wild turkey (*Meleagris gallopavo silvestris*) broods as they aged from day 1 to 28. Because broods become more plastic in behaviors once they begin roosting off the ground, we separated data into broods that were ground roosting (1–13 days) and tree roosting (14–28 days). We used Hidden Markov Models to identify 2 behavioral states (restricted and mobile). We extracted state-specific recursive movements based on states and specific step lengths, which we integrated into a step selection analysis to evaluate resource selection. We found that in a restricted state, ground roosting broods spent less time in areas of mixed pine-hardwoods and more time in areas with greater vegetation density. Tree roosting broods revisited areas closer to shrub/scrub landcover types, and areas with greater vegetation density. Tree roosting broods also spent less time near mixed pine-hardwoods, while spending more time in areas with greater vegetation density. We found that in a mobile state, ground roosting broods revisited areas closer to secondary roads and mixed pine-hardwoods, but farther from hardwoods. Tree roosting broods revisited areas farther from secondary roads and with greater vegetation density. Tree roosting broods also spent more time in areas closer to pine. Resource selection varied depending on behavioral state and recursive movements. However, revisitation and residence time impacted selection in both ground and tree roosting broods. Our findings highlight the need to consider how behaviors can influence movement decisions and ultimately resource selection.

## Introduction

Spatial distribution of resources such as forage, water, and shelter influences how animals move across the landscape^1,2^. Animals collect resources from patches within home ranges through repeated visitation^3–5^. By revisiting areas that contain resources, animals minimize risks associated with navigating unfamiliar areas which may improve survival and fitness^6–8^. Recursive movement patterns are returns to previously visited areas, and are a widespread phenomenon in the animal kingdom^9^. Understanding how recursive movement strategies influence behavioral processes is important in examining areas for resource acquisition^9–12^.

Recursive movement strategies have been documented in a variety of species, typically occurring when individuals are locating resources within a heterogeneous landscape^13–15^. Path recursions, defined as nonrandom movements in which animals repeatedly return to resource rich locations^9^, is a profitable foraging strategy that enables resources to recover^2,13^. Although generality of recursive movements is recognized in how animals select habitat, resource selection analyses often associate movements to availability of resources within individual home ranges^16–18^. Including recursion information within a resource selection framework can potentially identify one mechanism driving behavioral decision-making and resource availability within a home range^19,20^.

Behavioral decisions can influence the fitness of a species via resource selection^21–23^. Changes in behavior and movement patterns may suggest a response to variation in habitat conditions, such as where an individual goes to acquire resources or avoid risk^24,25^. Identifying behavioral patterns in resource selection could provide insight as to where individuals choose to travel versus forage^26–28^. Failing to incorporate such behavioral patterns in resource selection models can result in biased results, including misidentifying where animals travel and misallocating limits in foraging resources^29–31^.

Wild turkeys (*Meleagris gallopavo*) are considered habitat generalists; however, habitat requirements of adults differ from their precocial offspring^32^. During the first 28 days post-hatch, wild turkey poults experience high mortality risk^33–35^ as they are unable to thermoregulate and must find high quality foraging patches rich in arthropods^36–39^. Wild turkey poults grow rapidly during the first month, developing an ability to fly within 2 weeks post-hatch^40–42^. Increasing maneuverability facilitates behavioral changes and can alter foraging strategies to exploit areas within their range more efficiently^40,41^. Hence, resource needs and selection of poults may be more specialized immediately post-hatch and become more generalized as they age^35,39^.

Wild turkey broods are thought to revisit profitable areas^32,43^, but whether there are patterns in revisitations remains unknown. Knowledge of revisitation timing to specific sites across the landscape could provide insight to drivers of the selection processes used by broods^44^. Therefore, we examined how behavioral state-dependent recursive movements influenced resource selection as broods aged. Our objective was to quantify differences in recursive movements and resource selection of ground versus tree-roosting broods. We used movement behaviors to infer a behavioral state (mobile and restricted) to account for differences in resource selection. We hypothesized that broods would exhibit differential resource selection across behavioral states. We predicted that as broods aged, they would become more plastic in resource selection, but continue to exhibit consistency in recursive movements.

## Methods

### Capture and handling

We used rocket nets to capture wild turkeys from January-March of 2014–2021 (For details on study sites refer to Supplementary Information). We aged captured individuals based on presence of barring on the ninth and tenth primary feathers, and identified sex of each individual by the coloration of the breast feathers^42^. We banded each bird with an aluminum rivet leg band (National Band and Tag Company, Newport, Kentucky; female size = 8, male size = 9) and radio-tagged each individual with a backpack-style GPS-VHF transmitter^45^ produced by Biotrack Ltd. (Wareham, Dorset, UK). We programmed transmitters to record 1 GPS location nightly (23:58:58) and hourly GPS locations from 0500 to 2000 (Standard Time according to the appropriate time zones) until the battery died or the unit was recovered^46^. Each transmitter had a mortality switch that was programmed to activate after > 23 h of no movement. We released turkeys immediately at the capture location after processing.

### Nest and brood monitoring

We located wild turkeys ≥ 2 times per week using a 3-element handheld Yagi antenna and receiver to monitor survival based on the presence of a mortality signal, general movements of individuals across their ranges, and nesting activity. We remotely downloaded GPS locations from each turkey ≥ 1 time per week. In ArcGIS 10.8 (Environment Systems Research Institute, Redlands, California, USA), we spatially projected GPS locations to identify nest locations by determining when a female’s locations became concentrated, which represented the onset of incubation^47,48^. When GPS locations indicated nest termination, we located the nest site to determine if hatching had occurred^49–51^.

Following methods outlined by Chamberlain et al.^35^, we monitored brooding females until brood failure or 28 days after hatch, as most brood mortality occurs during this period^33^. We located females that hatched successfully every 2–3 days post-hatch via VHF to conduct brood surveys, and considered a brood to be present if ≥ 1 poult was seen or heard with the female^35^. If we detected a brooding female on the ground prior to sunrise less than 14 days post-hatch, we assumed she was still with a brood as brooding females typically begin tree roosting with poults 14 days post-hatch^32,34,40^. Hence, if we were able to detect a brood during the night, we did not disturb them during the day. Likewise, if we detected a brooding female roosted in a tree prior to 14 days post-hatch and could not detect poults, we assumed the brood was lost. We performed brood surveys up to 28 days after hatch or until we failed to detect any poults during 2 consecutive attempts, at which point we assumed the brood was lost. We defined brood success as the proportion of broods with ≥ 1 poult surviving to 28 days post-hatch^35^.

### GPS data processing

We processed and cleaned the GPS data by removing fix locations that had dilution of precision values (DOP) > 7. We excluded from analyses females that successfully hatched a nest but were never visually confirmed to have poults. We then removed roosting locations (1 point collected at midnight, 0500 and 0600) as we expected broods would rarely revisit roost sites^35,52^, and our interest was in behaviors most likely to be associated with foraging, loafing, or traveling.

### Behavioral analysis

**Figure 1:**
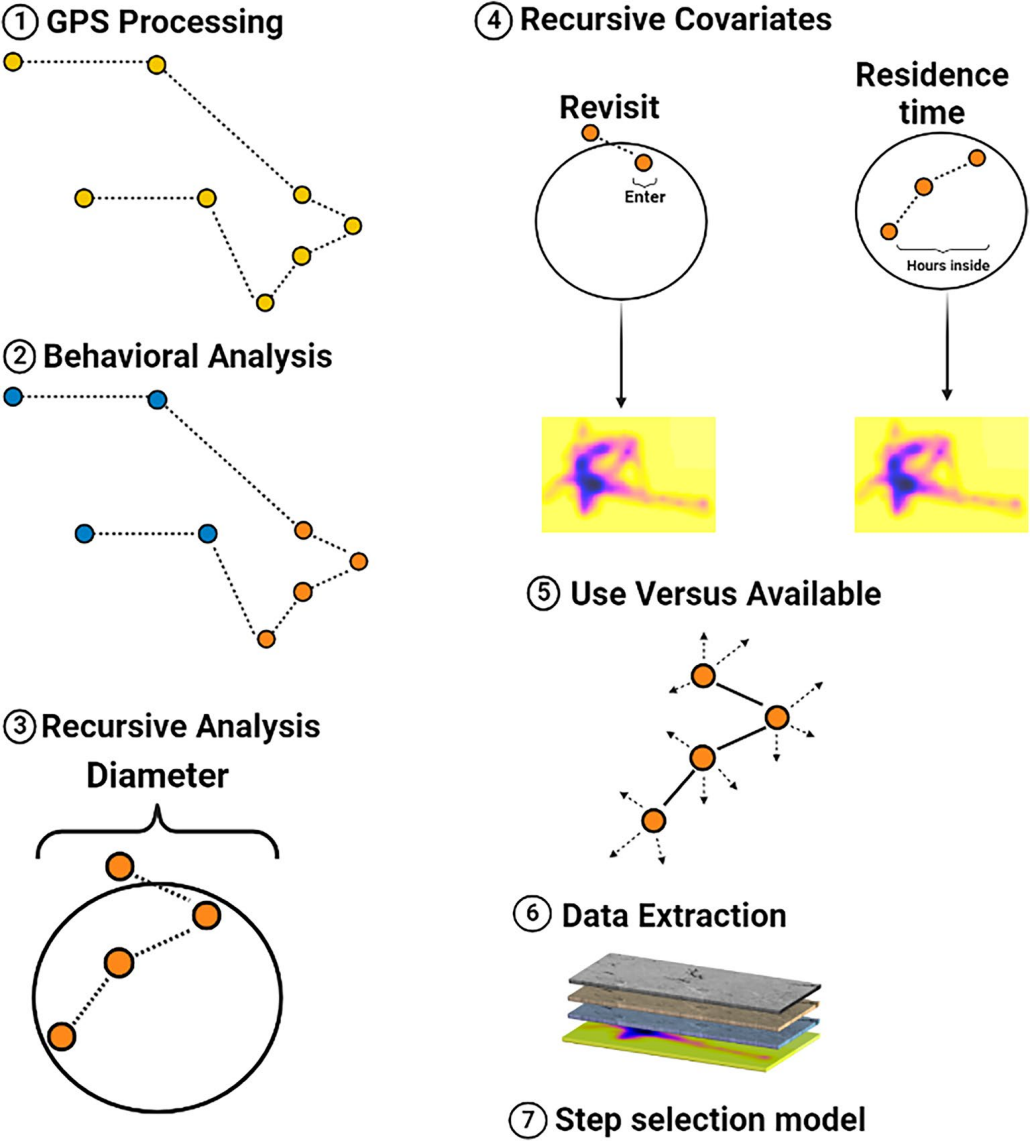
Conceptual framework for evaluating resource selection as a response to behavioral dependent recursive movements. This approach relies on standardizing movement data (1) that are used within a hidden Markov model to classify behavioral states (blue: mobile, orange: restrictive; 2). We then used each behavioral state to identify recursive movements based on a diameter appropriate to each behavioral state (star represents the GPS location of interest; 3) and created 2 raster layers representing revisit and residence time (4). We identified used versus available points based on step length and turn angle (5) and extracted environmental and recursive movement covariates (6). Finally, are data was fitted within a step selection function (7) to quantify in resource selection as broods aged.

We fit a Hidden Markov Model (HMM;^53^) to define movement trajectories into behavioral states based on step lengths and turning angles (Fig. 1). We modeled step lengths using a gamma distribution and turning angles using a Von Mises distribution^53^. We defined initial parameter values for 3 states: a stationary movement state with small step lengths (gamma distribution with a mean of 27 m and standard deviation of 27 m) and uniform turning angles (Von Mises distribution with a mean of π and a concentration of 0.1), a restricted movement state with small/moderate step lengths (gamma distribution with a mean of 150 m and standard deviation of 150 m) and uniform turning angles (Von Mises distribution with a mean of 2.5 and a concentration of 0.5), and a travelling movement state with large step lengths (gamma distribution with a mean of 400 m and a standard deviation of 1000 m) and directed turning angles (Von Mises distribution with a mean of 0.001 and a concentration of 0.99). We used the Viterbi algorithm to assign each step to the most likely behavioral state based on results of the HMM^54^. We conducted our analysis in R (v.4.1.0; R Core Team, 2022) using package momentuHMM^55^.

### Recursive analysis

Following Bracis et al.^44^, we calculated the revisit rate (hereafter, revisitation) as the sum of visits to previously visited locations as follows. We first assigned a unique identification to each female GPS location for the duration of known brooding. To quantify behavioral-specific revisitation, we buffered GPS locations using the mean step-length from our HMMs for restricted and mobile movements (90-m radius and 250-m radius) to identify the area likely used by each brood each hour (Fig. 1). We considered revisits to be GPS locations that fell within a spatial buffer for any previous day for the entire period the brood was monitored. Additionally, we calculated residence time, defined as the total elapsed time between successive GPS locations within the circular buffer for all visits during the observed observation period for each brood (i.e., up to 28 days), and return time, defined as the amount of time (days)between visits using the R package recurse^44^. For the recursion analyses, we evaluated brooding females independently of one another.

To incorporate the effect of recursive movements on resource selection, we used a modified Brownian bridge approach, the Biased Random Bridge kernel utilization^56,57^. We produced a 30-m by 30-m raster to represent behavioral-specific recursive movements for each individual (Fig. 1). We buffered GPS locations identified from our HMMs as restricted or mobile, using either the 90-m or 250-m radius as noted above. We created a 2-dimensional utilization distribution of each individual’s trajectory that represented the relative number of revisits made to each location^58^. We also assessed the time that a female spent in each area, which provided a biologically relevant measure of intensity of use. Specifically, we used Biased Random Bridge kernel utilization distributions based on residence time to evaluate intensity of use. We converted each behavioral-specific recursion map to a continuous value between 0 and 100, where 0 identified areas that were most strongly associated with the recursive behavior, and values around 100 identified areas not associated with recursive behaviors. We created recursive movement maps using the R package adehabitatHR^59^.

### Environmental covariates

We examined resource selection in relation to a set of environmental covariates relevant to ecology of brooding female wild turkeys. We obtained year-specific, 30-m resolution spatial data on landcover from the Cropland Data Layer (Cropscape) provided by the National Agricultural Statistics Service (National Agricultural Statistics Service 2015). We recoded and combined landcover in R to create 7 unique landcover types (water, pine forest, hardwood forest, mixed pine-hardwood forest, open treeless areas, shrub/scrub, and infrastructure). We then calculated the Euclidean distance in ArcMap 10.8 (Esri, Redlands, CA, USA) to get the distance a GPS location was located from each landcover type. We used 30‐m resolution imagery from United States Geological Survey (USGS) Landsat–8 Operational Land Imager to compute a normalized difference vegetation index (NDVI) in ArcMap 10.8 (Esri, Redlands, CA, USA) as an index of vegetation density^60^. Measurements of NDVI allowed us to understand sparseness of vegetation, which has been shown to influence maneuverability, concealment, and foraging ability for poults^36,41^. We used the USGS National Transportation Dataset (https://nationalmap.gov/transport.html) and information provided by the Department of Defense to obtain secondary road layers.

### Habitat and model selection

Before analysis, we scaled and centered all variables, so that we could compare effect sizes of variables within individuals on each respective study sites^61^. We tested for correlation among all continuous predictor variables using Pearson’s correlation and none were highly correlated (correlation coefficient > 0.7). We used a step selection function (SSF;^62,63^) to assess resource selection, where available habitat associated with a given female location was conditional on where the individual occurred at the time of the previous GPS location. We considered a used point as the GPS location of a female, whereas available points were 100 locations that were theoretically available for selection by that female during the hour the GPS location was recorded. We generated available locations using the amt package in R^64^. To assess temporal resource selection and recursive movement behaviors, we separated data into 2 periods based on whether females with broods were ground (day 1–13) or tree roosting (day 14–28). For each behavioral state (restricted or mobile), we then parameterized 6 models separately. Within each model, we included the logarithm of step length and the cosine of the turning angle as covariates to account for the underlying movement process^65^. Furthermore, we parameterized a landcover model, which included only landcover and secondary roads. We parameterized 2 models, one of which was based on number of revisits only, and the other based on only residence time. Finally, we parameterized 2 models that contained all covariates and which interacted with either number of revisits or residence time (composite model; Table 1).

**Table 1:** Model structure for composite, landcover, and recursive models for ground and tree roosting eastern wild turkey (*Meleagris gallopavo silvestris*) broods during 2014–2021 across 11 study sites distributed throughout the southeastern United States. Each resource covariate was calculated as a distance (m) to metric.

Model	Parameter
Ground roosting	
Restricted state	
Revisit	
Landcover	Secondary roads + hardwoods + mixed pine-hardwoods + normalized difference vegetation index + open + pine + shrub/scrub + cosine of turn angle + logarithm of step length
Recursion-only	Revisitation
Composite	Landcover + recursion only + resource * recursion
Residence time	
Landcover	Secondary roads + hardwoods + mixed pine-hardwoods + normalized difference vegetation index + open + pine + shrub/scrub + cosine of turn angle + logarithm of step length
Recursion-only	Residence time
Composite	Landcover + recursion only + resource * recursion
Mobile state	
Revisit	
Landcover	Secondary roads + hardwoods + mixed pine-hardwoods + normalized difference vegetation index + open + pine + shrub/scrub + Cosine of turn angle + logarithm of step length
Recursion-only	Revisitation
Composite	Landcover + recursion only + resource * recursion
Residence time	
Landcover	Secondary roads + hardwoods + mixed pine-hardwoods + normalized difference vegetation index + open + pine + shrub/scrub + Cosine of turn angle + logarithm of step length
Recursion-only	Residence time
Compsite	Landcover + recursion only + resource * recursion
Tree roosting	
Restricted state	
Revisit	
Landcover	Secondary roads + hardwoods + mixed pine-hardwoods + normalized difference vegetation index + open + pine + shrub/scrub + cosine of turn angle + logarithm of step length
Recursion-only	Revisitation
Composite	Resource only + recursion only + resource * recursion
Residence time	
Landcover	Secondary roads + hardwoods + mixed pine-hardwoods + normalized difference vegetation index + open + pine + shrub/scrub + Cosine of turn angle + logarithm of step length
Recursion-only	Residence time
Composite	Landcover + recursion only + resource * recursion
Mobile state	
Revisit	
Landcover	Secondary roads + hardwoods + mixed pine-hardwoods + normalized difference vegetation index + open + pine + shrub/scrub + cosine of turn angle + logarithm of step length
Recursion-only	Revisitation
Composite	Landcover + recursion only + resource * recursion
Residence time	
Landcover	Secondary roads + hardwoods + mixed pine-hardwoods + normalized difference vegetation index + open + pine + shrub/scrub + cosine of turn angle + logarithm of step length
Recursion-only	Residence time
Composite	Landcover + recursion only + resource * recursion

We used mixed conditional Poisson regression models with stratum-specific intercepts to estimate resource selection^63^. To account for variability among individuals within our models, we included random slope for each covariate for each unique individual^66^. We did not include random slopes for each interaction of landcover and recursive movement as models failed to converge due to quasi-complete separation. We fitted the SSF using the Poisson formulation where the stratum-specific random intercept variance was fixed to a large value to avoid shrinkage, following Muff et al.^63^. We used the R package glmmTMB to conduct the step selection analysis^67^.

We used second-order Akaike's Information Criteria (AIC*c*) to assess the amount of support for the different candidate models^68,69^. We calculated ΔAIC*c* values between the AIC*c* value for candidate model *i* and the lowest-ranked AIC value. We also calculated Akaike's weights (*w*_*i*_) for each model. We then calculated parameter estimates and their standard errors for all covariates in models within 2 ΔAIC*c* units of the lowest-ranked AIC value. To assess how well our SSF models explained the data, we used area under the receiver-operating characteristic curves (AUC) calculated with the pROC package^70^. An AUC value of 0.5 indicated the model provided estimates of no better than random predictions but values greater than 0.7 indicated a better model fit with more accurate predictions.

### Ethical approval

All methods were performed in accordance with the relevant guidelines including turkey capture, handling, and marking procedures which were approved by the Institutional Animal Care and Use Committee at the University of Georgia (Protocol #A2014 06008Y1A0, A343701, A2016 04-001-R1, A2019 01-025-R2, and A2020 06-018-R1) and the Louisiana State University Agricultural Center (Protocol #A2014-013, A2015-07 and A2018-13).

## Results

We captured and radio‐marked 663 female wild turkeys during 2014–2021. We monitored 692 nest attempts, 147 (21.2%) of which successfully hatched. We censored data from 10 broods that were presumably lost during or within hours of hatching, as we were not able to visually document poult presence via brood surveys. We censored an additional 26 broods due to GPS failure. Hence, we used 111 broods in our analyses, which we visually monitored until brood failure or 28 days after hatch. Of these 111 broods, 36 (32%) survived to 28 days post‐hatch. After removing roosting locations, we used 33,819 GPS locations to use for subsequent analyses.

### Behavioral classifications

**Figure 2:**
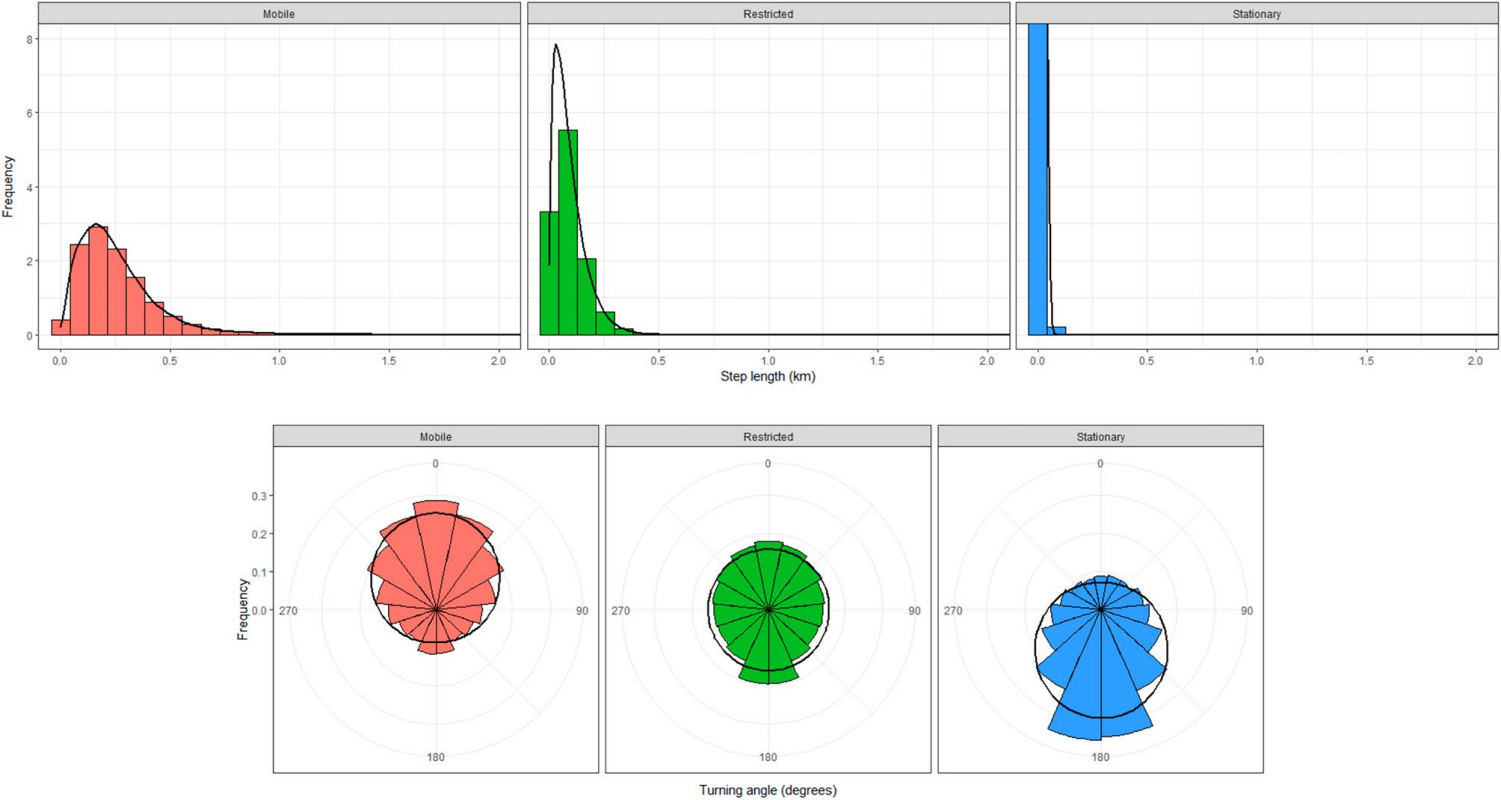
Step length and turning angle distributions for movements of eastern wild turkey (*Meleagris gallopavo silvestris*) broods across the southeastern United States during 2014–2021. The black curves depict distributions based on parameter values estimated with a Hidden Markov Model, which are overlaid on histograms of the raw data.

From our HMM, the step length distribution had an estimated mean of 13.3 m (95% CI = 13.2–13.4 m) and standard deviation of 10.1 m (95% CI = 10.0–10.2 m) for the stationary state, an estimated mean of 92.2 m (95% CI = 90.7–93.8 m) and standard deviation of 74.1 m (95% CI = 72.7–75.5 m) for the restricted state, and an estimated mean of 249.1 m (95% CI = 245.7–252.4 m) and standard deviation of 175.9 m (95% CI = 175.0–177.2 m) for the mobile state (Fig. 2). The turning angle distribution had an estimated mean of 3.1 (95% CI = 3.1–3.2) and concentration parameter of 0.7 (95% CI = 0.7–0.8) for the stationary state, a mean of 2.4 (95% CI = 0.7–3.1) and concentration parameter of 0.01 (95% CI = − 0.01 to 0.02) for the restricted state, and a mean of − 0.1 (95% CI = − 0.04 to 0.01) and concentration parameter of 0.5 (95% CI = 0.5–0.6) for the mobile state (Fig. 2). For subsequent analyses, we combined the stationary and restricted states as they were both associated with relatively shorter distances moved and sharper turn angles characteristic of foraging bouts. From the HMM, we considered 84.5% of movements restricted whereas 15.5% were mobile.

### Recursive movements and behavioral dependent resource selection

**Figure 3:**
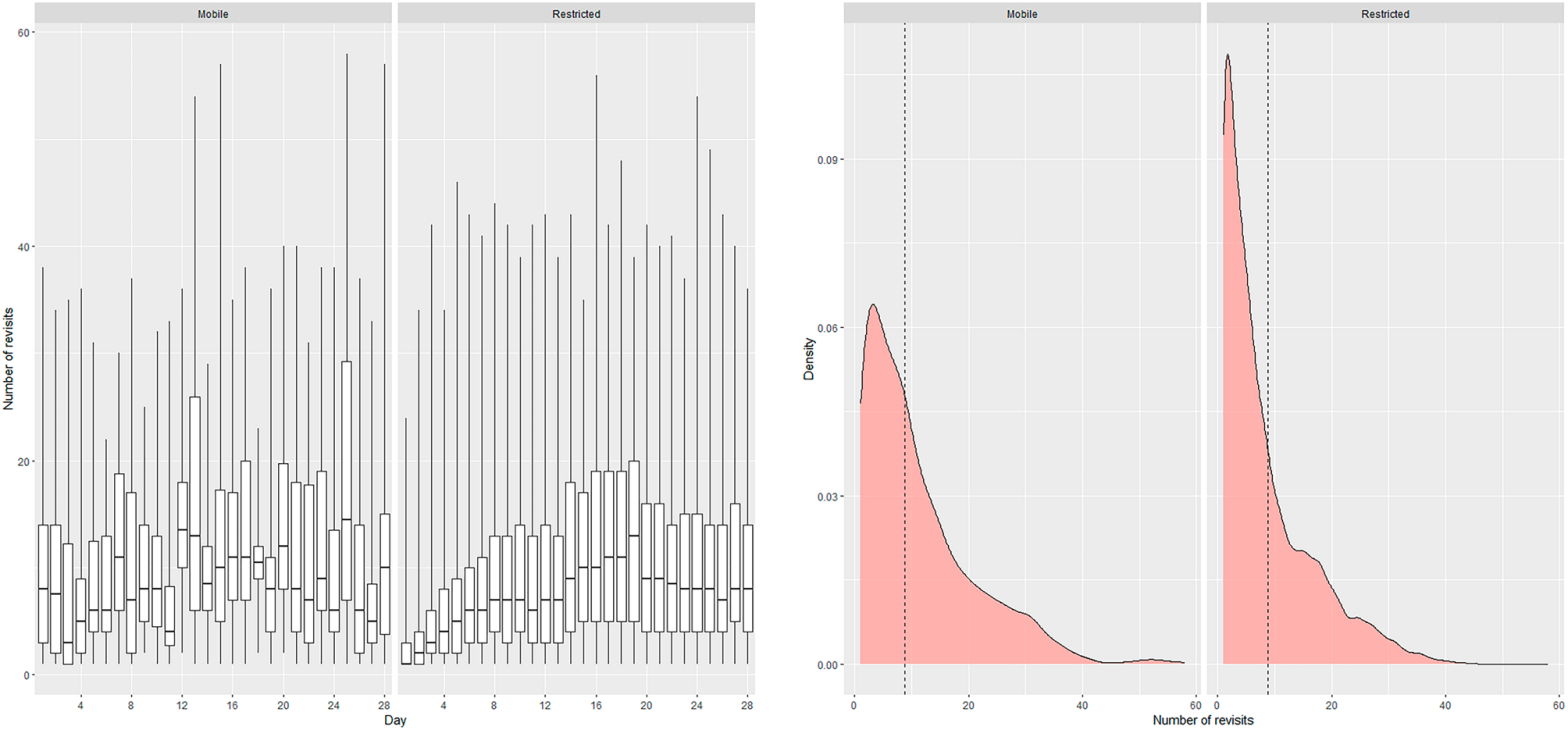
Boxplots of daily revisitation events by each behavioral state (mobile and restricted) and density plots showing the distribution of the data for eastern wild turkey (*Meleagris gallopavo silvestris*) broods across the southeastern United States during 2014–2021. The dashed black line in the density plot is the mean of the data.

**Figure 4:**
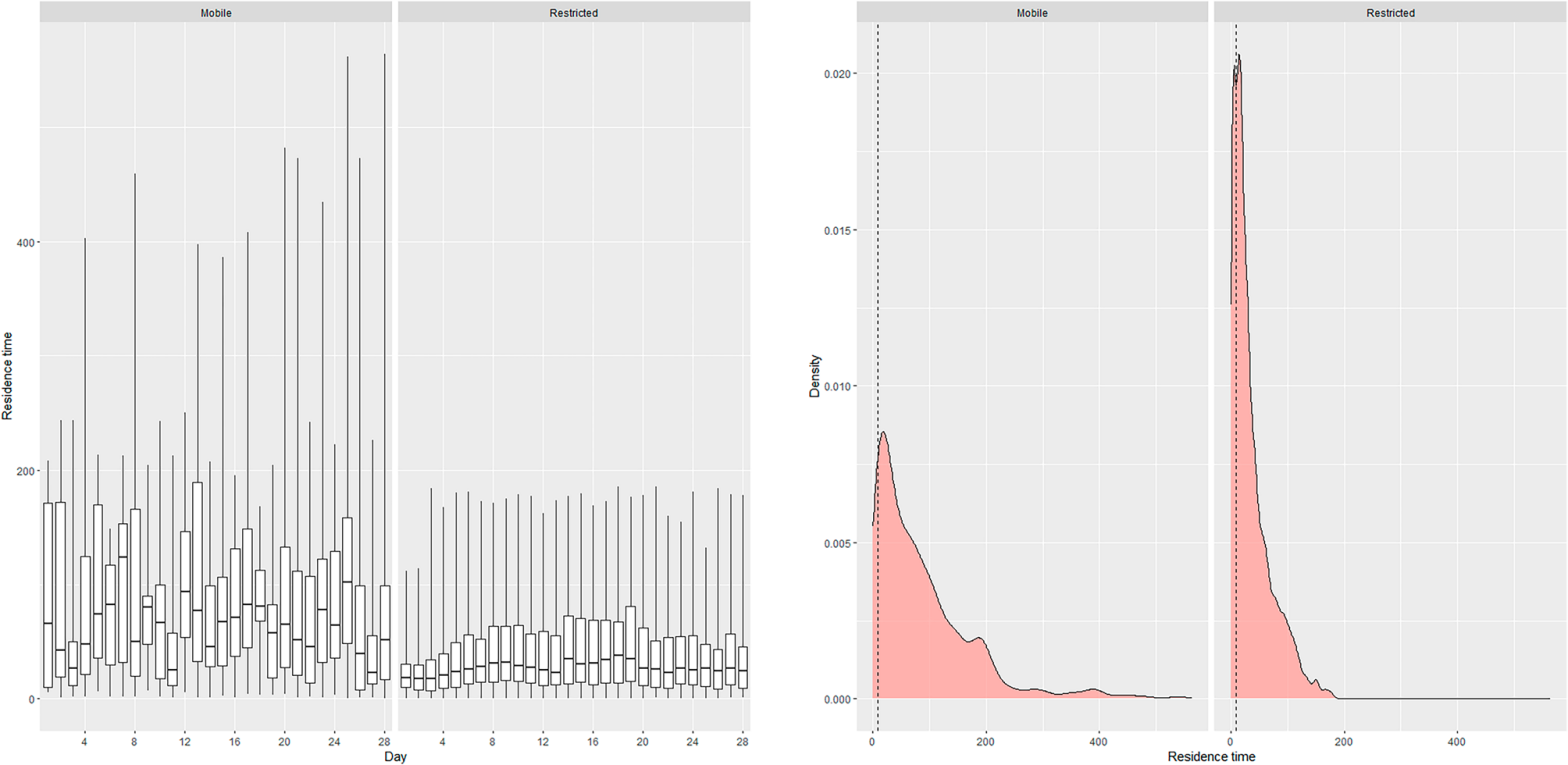
Boxplots of daily residence time by each behavioral state (mobile and restricted) and density plots showing the distribution of the data for eastern wild turkey (*Meleagris gallopavo silvestris*) broods across the southeastern United States during 2014–2021. The dashed black line in the density plot is the mean of the data.

**Figure 5:**
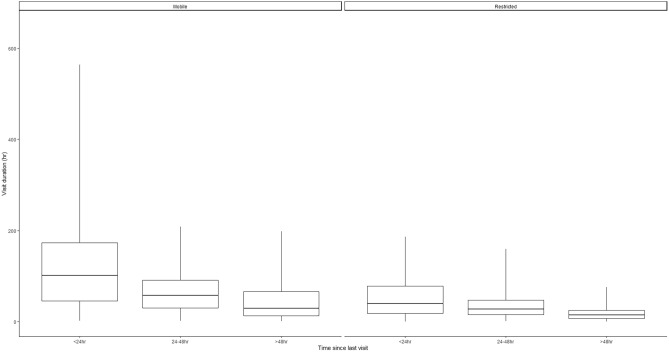
Boxplots of time since last visit by each behavioral state (mobile and restricted) of eastern wild turkey (*Meleagris gallopavo silvestris*) broods across the southeastern United States during 2014–2021.

On average, broods revisited previous locations 9.2 times (SD = 8.1, range = 0–58 visits; Fig. 3). Mean residence time was 43.2 h (SD = 47.3 h, range = 0.1–564.0 h; Fig. 4), whereas the return time averaged 1.4 days (SD = 2.2, range = 0–25.9 days; Fig. 5).

**Figure 6:**
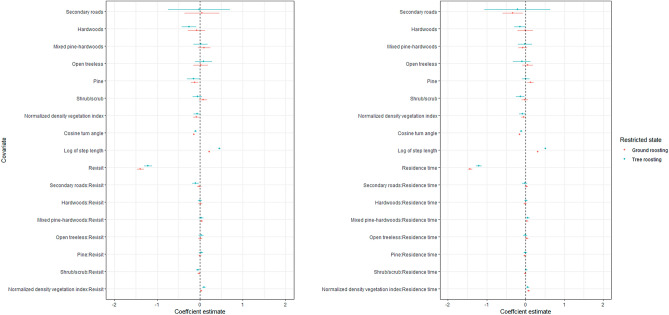
Coefficient plot depicting habitat selection of eastern wild turkey (*Meleagris gallopavo silvestris*) broods across the southeastern United States during 2014–2021 while in a restricted behavioral state. The left plot refers to revisit composite model while the right corresponds to the residence composite model. The whiskers depict 95% confidence intervals around mean estimates.

**Figure 7:**
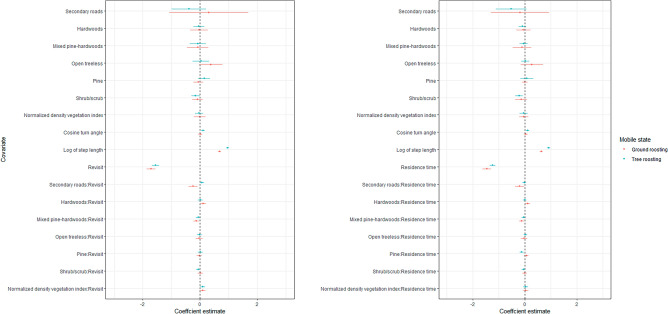
Coefficient plot depicting habitat selection of eastern wild turkey (*Meleagris gallopavo silvestris*) broods across the southeastern United States during 2014–2021 while in a mobile behavioral state. The left plot refers to revisit composite model while the right corresponds to the residence composite model. The whiskers depict 95% confidence intervals around mean estimates.

The composite models that included both recursive movements and landcover performed better than those based exclusively on landcover or recursive movements (Table 2). Within each model broods revisited and exhibited increased residence time regardless of landcover (Figs. 6, 7). Ground roosting broods in a restricted state spent less time in mixed pine-hardwoods (β = 0.04, 95% CI = 0.00–0.07) and more time at locations with greater vegetation density (β = 0.08, 95% CI = 0.04–0.1). Ground roosting broods selected locations closer to secondary roads (β = − 0.33, 95% CI = − 0.59 to − 0.07) but did not revisit or spend time in these areas. Ground roosting broods selected areas farther from pine forest (β = 0.1, 95% CI = 0.04–0.2).

**Table 2:** Akaike’s Information Criterion with small sample bias adjustment (AIC_c_), number of parameters (*K*), ΔAIC_c_, adjusted Akaike weight of evidence (w_i_) in support of model, log-likelihood (LL), and area under the receiver-operating characteristic curves (AUC) for each final model examining habitat selection within 2 behavioral states by ground and tree roosting eastern wild turkey (*Meleagris gallopavo silvestris*) broods at 11 sites across the southeastern United States during 2014–2021.

Model	*K*	AIC_c_	ΔAIC_c_	w_i_	LL	AUC
Ground roosting						
Restricted state						
Revisit						
Composite	25	298,919.7	0.0	1.0	− 149,434.8	0.73
Recursion-only	4	300,157.0	1237.3	0.0	− 150,074.5	0.71
Landcover	16	309,947.2	11,027.6	0.0	− 154,957.6	0.61
Residence time						
Composite	25	295,362.2	0.0	1.0	− 147,656.1	0.76
Recursion-only	4	296,202.6	840.4	0.0	− 148,097.3	0.74
Landcover	16	309,955.0	14,592.8	0.0	− 154,961.5	0.61
Mobile state						
Revisit						
Composite	25	35,555.5	0.0	1.0	− 17,752.72	0.82
Recursion-only	4	35,693.7	138.2	0.0	− 17,842.8	0.80
Landcover	16	37,618.9	2063.5	0.0	− 18,793.5	0.69
Residence time						
Composite	25	36,061.9	0.0	1.0	− 18,006.0	0.80
Recursion-only	4	36,145.7	83.8	0.0	− 18,068.9	0.78
Landcover	16	37,618.9	1557.0	0.0	− 18,793.5	0.69
Tree roosting						
Restricted state						
Revisit						
Composite	25	170,671.7	0.0	1.0	− 85.310.8	0.73
Recursion-only	4	171,002.5	330.8	0.0	− 85,497.3	0.72
Landcover	16	176,391.2	5719.5	0.0	− 88,179.6	0.62
Residence time						
Composite	25	169,489.7	0.0	1.0	− 84,719.9	0.75
Recursion-only	4	169,965.3	475.6	0.0	− 84,978.6	0.74
Landcover	16	176,391.2	6901.4	0.0	− 88,179.6	0.62
Mobile state						
Revisit						
Composite	25	39,078.4	0.0	1.0	− 19,514.2	0.82
Recursion-only	4	39,136.4	58.0	0.0	− 19,564.2	0.81
Landcover	16	41,245.0	2166.6	0.0	− 20,606.5	0.69
Residence time						
Composite	25	39,508.7	0.0	1.0	− 19,729.3	0.80
Recursion-only	4	39,587.1	78.4	0.0	− 19,789.5	0.79
Landcover	16	41,245.0	1736.3	0.0	− 20,606.5	0.69

When in a mobile state, ground roosting broods in a mobile state revisited and spent more time at locations closer to secondary roads (β =  − 0.2, 95% CI = − 0.4 to − 0.1), and mixed pine-hardwoods (β =  − 0.1, 95% CI = − 0.2 to − 0.02), but farther from hardwoods (β = 0.1, 95% CI = 0.02–0.21; Fig. 6). Tree roosting broods revisited areas closer to secondary roads (β =  − 0.1, 95% CI = − 0.2 to − 0.03), and shrub/scrub (β =  − 0.05, 95% CI = − 0.1 to − 0.01), and with greater vegetation density (β = 0.09, 95% CI = 0.05–0.1) while in a restricted state. Likewise, tree roosting broods in a restricted state spent less time near mixed pine-hardwoods (β = 0.05, 95% CI = 0.005–0.1), but more time in areas with greater vegetation density (β = 0.05, 95% CI = 0.02–0.09). Tree roosting broods in a restricted state selected areas closer to hardwoods (β =  − 0.1, 95% CI = − 0.3 to − 0.003) and shrub/scrub (β =  − 0.1, 95% CI = − 0.2 to − 0.03), regardless of revisitation and residence time.

When in a mobile state, tree roosting broods revisited areas with greater vegetation density (β = 0.1, 95% CI = 0.007–0.2) while in a mobile state. Likewise, tree roosting broods spent more time in areas closer to pine forest (β =  − 0.1, 95% CI = − 0.2 to − 0.04). Tree roosting broods in a mobile state selected areas closer to shrub/scrub (β =  − 0.2, 95% CI = − 0.4 to − 0.07), regardless of revisitation and residence time.

## Discussion

We found that resource selection of wild turkey broods varied depending on behavioral state and recursive movements. Further, we noted that resource selection differed for broods that roosted on the ground (1–14 days old) versus those that roosted off the ground (15–28 days old), although broods exhibited consistent recursive movements regardless of their age. Our approach allowed us to integrate movement behaviors into an improved understanding of resource selection^9,58,71^. Our SSF indicated that incorporating recursive movements with landcover improved model fit relative to standard SSF approaches, which typically only consider habitat variables and disregard movement behaviors that could influence selection^72,73^. Incorporating behavioral states that have potential to influence animal movements and decision-making can increase our ability to understand species movement ecology^74^.

Our behavioral analysis identified a restricted state characterized by shorter step lengths and less concentrated turning angles, and a mobile state characterized by longer step lengths and turning angles concentrated around zero, which was not surprising given similar findings in contemporary literature^22,75^. We observed that broods spent most of their time in a restricted rather than mobile state, consistent with Chamberlain et al.^35^. Restricted movements are often characterized as area-restricted search, loafing, or foraging behaviors^76,77^, whereas mobile movements are those such as walking, which represent directional movements away from a patch or along travel corridors^28,30^. We offer that ignoring behavioral states and how they influence movement could lead to misinterpretation of resource selection models. For instance, mobile movements occur at a much larger spatial scale than restricted movements as individuals are covering more area, and within our analysis we defined availability by step lengths of each state, making selection more representative of the behavior^22,62^. Overall, our findings indicate that recursive movements occur in each behavioral state, and resource selection differs by behavioral state whether individuals were ground or tree roosting.

Recursive movements are common across wildlife species^9^. We found recursive movements to be important on both a behavioral and temporal scale during brooding. Brooding females are faced with the challenge of finding quality foraging opportunities near vegetative cover that provides concealment^78,79^. Our results support the idea that wild turkey broods increased residence time at locations and develop behaviors that reflect area-restricted searching^35,43,80^. Area-restricted foraging presumably allows broods to limit movements and space use, which may positively influence foraging success and reduce predation risk^81–83^. Our results also indicate that recursive movements occur while broods are in a mobile state, suggesting broods were moving through familiar areas. Recursive movements to areas previously visited increases environmental predictability for individuals, which may increase fitness as individuals familiarize themselves with resources on the landscape^84^. Overall, our results suggest that broods were returning to and spending more time in specific locations, presumably to areas which increase maneuverability and foraging opportunities.

When animals are moving from one resource patch to the next, they may exhibit differential resource selection^85,86^. We observed differences in resource selection between broods that were ground versus tree roosting when they were in a mobile state. When in a mobile state, ground roosting broods were more likely to be closer to secondary roads and mixed pine-hardwoods, while avoiding areas closer to hardwoods. As broods aged and began to roost in trees, they selected for pine forest, shrub/scrub, and areas with increasing vegetation density. Traveling and feeding rates of brooding galliforms are impacted by the ability of individuals to maneuver through ground cover^36,87^. Thus, behavioral strategies in relation to vegetation composition is critical to brooding individuals and will depend on morphological development^32,88^. The differences we observed in resource selection as broods aged provide evidence that selection becomes more plastic as broods age.

In precocial birds, resource requirements and degree of resource specialization may vary due to body size that can influence mobility, thermoregulation, foraging, and responses to predation risk^89,90^. Our findings demonstrate that accounting for underlying behavior and temporal scales as broods age may change assessments of resource selection. For many species, broods are inextricably linked to early successional vegetation communities that offer quality foraging opportunities with reduced predation risk^88,91–93^. We observed that broods in a restricted behavioral state (i.e., foraging, loafing) selected secondary roads and areas with increased vegetation density during the first 14 days of life when they roosted on the ground. Conversely, after broods reached 14 days of age and began roosting in trees, they selected areas closer to shrub/scrub and hardwood landcover types, and areas with increased vegetation density when they were in a restricted behavioral state. Hence, broods exhibited rapid changes in behavioral plasticity as they aged, which would contribute to the temporal changes in resource selection we observed, and parallel similar observations in contemporary literature on wild turkey broods^35,39^. Increasing behavioral plasticity as broods age has been reported in other gallinaceous species, in that as broods age their diet breadth^88,94,95^ and mobility^87^ change, allowing them to exploit more profitable patches within their ranges.

### Supplementary Information

Supplementary Information.

## Data Availability

The datasets generated during and/or analyzed during the current study are available from the corresponding author on reasonable request.
